# UHMK1 Is a Novel Marker for Personalized Prediction of Pancreatic Cancer Prognosis

**DOI:** 10.3389/fonc.2022.834647

**Published:** 2022-03-10

**Authors:** Yiqiao Luo, Shanshan Han, Bin Yan, Huihui Ji, Lian Zhao, Jury Gladkich, Ingrid Herr

**Affiliations:** Molecular OncoSurgery, Section Surgical Research, Department of General, Visceral and Transplant Surgery, University of Heidelberg, Heidelberg, Germany

**Keywords:** pancreatic cancer, long noncoding RNA, UHMK1, tumor marker, nomogram

## Abstract

Pancreatic ductal adenocarcinoma (PDAC) is among the leading causes of cancer mortality, and new therapeutic options are urgently needed. Long noncoding RNA H19 (H19) is known to promote PDAC progression, but the downstream genes of H19 are largely unknown. Five PDAC cell lines, nonmalignant pancreatic cells, TCGA, GEO-derived pancreatic tissues (malignant, n=413; nonmalignant, n=234), a pancreatic tissue array (n=96), and pancreatic tissues from our clinic (malignant, n=20; nonmalignant, n=20) were examined by a gene array, RT-qPCR, Western blotting, MTT, colony formation, wound-healing, siRNA-mediated gene silencing, bioinformatics, xenotransplantation, and immunohistochemistry assays. The cell cycle inhibitor, UHMK1, was identified to have the strongest correlation with H19. UHMK1 expression was enhanced in PDAC, and high UHMK1 expression correlated with tumor stage, and lower overall survival. siRNA-mediated UHMK1 downregulation inhibited progression signaling. siRNA-mediated downregulation of H19 or UHMK1 inhibited tumor proliferation and xenograft growth. Based on the correlation between UHMK1 expression and clinical parameters, we developed a nomogram that reliably predicts patient prognosis and overall survival. Together, we characterized UHMK1 as an H19-induced oncogene and verified it as a novel PDAC prognostic marker for overall survival.

## Introduction

Pancreatic ductal adenocarcinoma (PDAC) is one of the leading causes of cancer mortality worldwide and is characterized by late diagnosis, early metastasis, and high therapy resistance (1). Despite worldwide efforts, therapeutic options for PDAC are limited (2, 3), and improvement is urgently needed.

Long noncoding RNAs (lncRNAs) are noncoding RNAs that contain more than 200 nucleotides (4). LncRNAs epigenetically regulate gene expression by modulating transcriptional activities, posttranscriptional activities, genomic imprinting, and other biological processes (5). Recently, the lncRNA H19 (H19) has been identified as a cancer promotor in different cancer types (6–9). H19 is highly expressed in PDAC, and it promotes proliferation, migration, and metastasis (6, 10–12). We identified the innate anti-viral immunity gene APOBEC3G as a major H19 downstream gene (12, 13) and demonstrated that the downregulation of H19 or APOBEC3G by siRNA or the bioactive agent sulforaphane prevented H19-mediated PDAC progression features as demonstrated by assays for colony formation, migration, invasion, Smad2 phosphorylation and tumor xenograft growth (12). Nevertheless, the function of additional, yet unknown, H19 target genes needs to be clarified.

The U2AF homology motif kinase 1 (UHMK1) was initially identified as a regulator of cyclin-dependent kinase inhibitors and the cell cycle regulator, p27 (Kip1) (14). Moreover, a more ubiquitous role of UHMK1 in cellular signaling is known, e.g., as a regulator of splicing factors 1 (15, 16), and RNA-binding proteins (17). Recently, a function of UHMK1 in the progression of hepatocellular carcinoma (18), gastric cancer (19), and ovarian cancer (20) has been reported, but the function of UHMK1 in PDAC is unclear.

Here, we demonstrated a high correlation between H19 and UHMK1, because the siRNA-mediated downregulation of H19 resulted in strong inhibition of UHMK1 RNA and protein expression. We further explored the role of UHMK1 in PDAC and found that high UHMK1 expression correlated with a shorter overall survival of PDAC patients. siRNA-mediated knockdown of UHMK1 expression was associated with reduced viability, clonogenicity, and migration. The inhibition of both H19 and UHMK1 prevented PDAC xenograft growth. Using UHMK1 expression and clinical data, we constructed a prognostic nomogram with high accuracy, which provides a new clinical tool to predict the prognosis of PDAC patients and aid in the treatment decision-making process.

## Results

### UHMK1 Is Highly Expressed in PDAC, Which Can be Inhibited by SiRNA-Mediated Downregulation of H19

**Figure 1:**
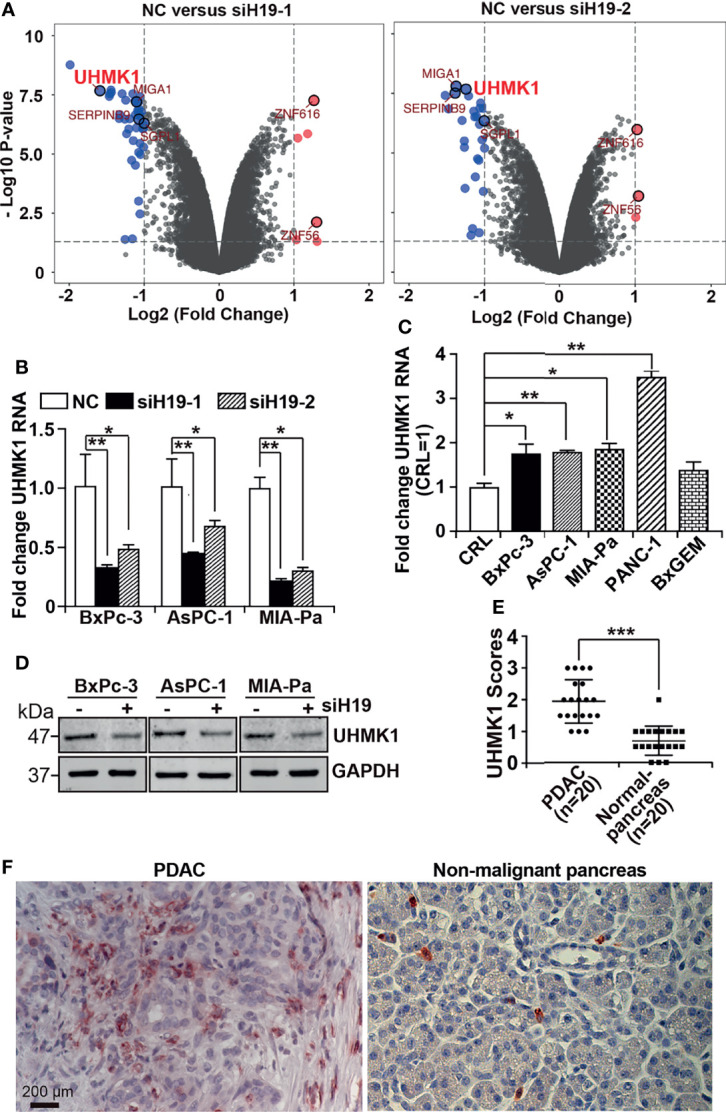
LncRNA H19-induced UHMK1 is highly expressed in PDAC. **(A)** Volcano plot showing differentially expressed genes in MIA-PaCa2 cell line depleted for lncRNA H19 using the siRNAs siH19-1 (left panel) and siRNAs siH19-2 (right panel), in comparison with cells transfected with a nonsense siRNA control (NC). The threshold was set to a log_2_ (fold change) >1 and a P value <0.05. Vertical axis corresponds to the statistical significance level provided as the -Log10 P value. The horizontal dashed gray line shows the P-value cutoff (-log10 1.3 ≙ P = 0.05) with points above the line having P values <0.05. The vertical gray dashed line indicates 1-fold changes/doubling (log2-fold change of 1), The six most significantly differentially regulated genes were MIGA1, SERPINB9, SGPL1, ZNF616, ZNF56, and UHMK1. **(B)** UHMK1 mRNA expression in the PDAC cell lines BxPc-3, AsPC-1 and MIA-PaCa2 after depletion of lncRNA H19, compared with control cells (NC). **(C)** UHMK1 mRNA expression in CRL-4023 (CRL), and the PDAC cell lines BxPc-3, AsPC-1, MIA-PaCa2, PANC-1, and BxGEM, The data were normalized to the expression of CRL-4023 cells. **(D)** GAPDH served as a loading control. The protein sizes in kilodaltons (kDa) are shown on the right. The crude Western blot images are shown in Figure S2. **(E)** UHMK1 protein expression was detected in paraffin-embedded human tissue derived from PDAC (n = 20) or nonmalignant (normal) pancreata (n = 20) by performing immunohistochemistry. Cell nuclei were stained with hematoxylin. **(F)** Representative images of UHMK1 expression in PDAC and nonmalignant pancreatic tissues are shown. The scale bar indicates 200 µm. *P < 0.05, **P < 0.01, and ***P < 0.001.

Recently it was shown that H19 is highly expressed in PDAC and we demonstrated that the siRNA-mediated downregulation of H19 inhibited progression features of PDAC (10, 12). To further investigate these promising results, the aim of the present study was to identify H19 mediators and to explore their function in PDAC progression. H19 expression was inhibited in MIA-PaCa2 cells by lipotransfection of two H19 siRNA constructs, whose functionalities were recently confirmed (12), along with a nonsense siRNA control. RNA was isolated 24 h after transfection, and gene array analysis was performed. Bioinformatics evaluation revealed 49 differentially expressed genes for siH19-1 and 30 differentially expressed genes for siH19-2 (**Supplementary Figures S1A, B**). Using the UpSetR R package, we selected six candidate genes associated with siH19-1 and siH19-2 as shown by volcano plots (**Figure 1A**) and a Venn diagram (**Supplementary Figure S1C**). At the top of the H19-downregulated candidate genes was UHMK1, closely followed by MIGA1, SERPINB9, and SGPL1, whereas ZNF56 and ZNF616 were upregulated by H19. Based on the online database TIMER 2.0, Pearson correlation analysis detected a positive correlation between UHMK1 and SGPL1, SERPINB9, and MIGA1 with R=0.524, R=0.296, and R=0.798, respectively, as presented by dot plots (**Supplementary Figure S2A**). By utilizing GEPIA online database, we figured out that each of these genes was significantly upregulated in PDAC patient tissues compared to nonmalignant pancreatic tissues (**Supplementary Figure S2B**). These results suggested that H19 drives the progression of PDAC not only through UHMK1 but also through several downstream genes simultaneously.

To verify these results, we lipotransfected siH19-1 and siH19-2 along with a nonsense siRNA control into BxPc-3, AsPC-1, and MIA-PaCa2 cells. After 24 h, total RNA was extracted, and UHMK1 expression was examined by RT-qPCR. The RNA expression of UHMK1 was significantly downregulated after knockdown by both siH19 constructs in all cell lines examined (**Figure 1B**). Because siH19-1 was most potent in the downregulation of UHMK1 mRNA expression, we used siH19 for all subsequent experiments. Next, we studied UHMK1 mRNA expression by RT-qPCR in the nonmalignant pancreas cell line, CRL-4023, and five PDAC cell lines. Compared to CRL-4023 cells, there was significantly increased UHMK1 expression in four of the five PDAC cell lines (**Figure 1C**). We confirmed that knockdown of H19 decreased UHMK1 expression in protein level by Western blot analysis (**Figure 1D** and **Supplementary Figure S3**). To evaluate UHMK1 protein expression in PDAC tissue from patients, we performed immunohistochemistry on PDAC tissues (n=20) and nonmalignant pancreatic tissues (n=20), which were obtained from brain-dead donors (**Table S1**). The expression level of UHMK1 was quantified by counting the percentage of UHMK1-positive cells of 10 randomly chosen vision fields of each tissue by two independent researchers with experience in pancreas histology who were blinded to the conditions. High, medium, low, and no expression was scored as 3, 2, 1, and 0, respectively. We discovered higher UHMK1 expression in PDAC tissues compared to nonmalignant, inflamed pancreatic tissue (**Figure 1E**), which can be seem in the representative staining (**Figure 1F**).

### Increased UHMK1 Expression Correlates With the Clinical Stage of PDAC

**Figure 2:**
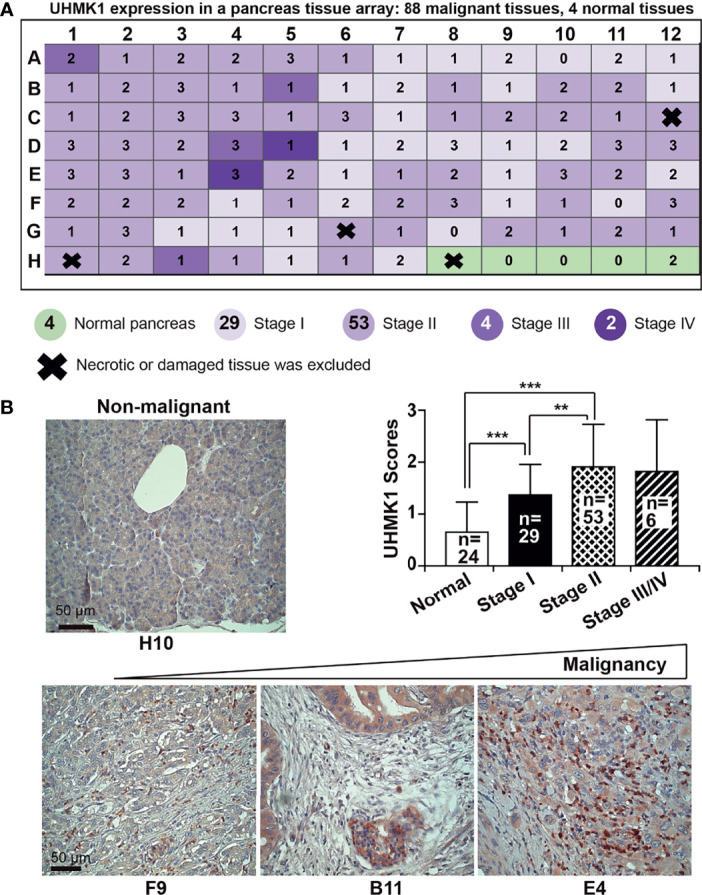
UHMK1 expression is related to PDAC tumor stage. **(A)** UHMK1 expression was examined by immunohistochemical staining of a commercially available PDAC tissue microarray with paraffin-embedded pancreatic tissue, which contained 91 malignant tissues from stage I to IV and 5 normal pancreatic tissues. Four tissues of the microarray were necrotic or damaged and were therefore excluded from the evaluation, and these tissues are indicated by thick black crosses on the schematic. The expression of UHMK1 was evaluated by immunohistochemistry under 400× magnification. The expression level was quantified by counting the positive, dark-brown cells per tissue by two independent researchers with expertise in pancreas histology who were blinded by the conditions. UHMKI expression was scored based on the following scale: high UHMK1 expression, 3; medium UHMK1 expression, 2; low UHMK1 expression, 1; and no UHMK1 expression, 0. **(B)** Representative images of UHMK1 expression in normal pancreatic tissue and in tissues of different PDAC stages are shown. Because there were only four nonnecrotic tissues from the nonmalignant pancreas available in this tissue array, we included data from the 20 previously examined nonmalignant pancreatic tissues. The mean expression of UHMK1 according to the previously scored values is shown. The scale bar indicates 50 µm. **P < 0.01, and ***P < 0.001.

To examine UHMK1 expression in different cancer stages, we performed immunohistochemistry using a commercially available pancreatic cancer tissue array with 91 malignant tissues and 5 nonmalignant pancreatic tissues along with patient information on clinical stage and pathology grade (**Table S2**). UHMK1-positive cells were quantified by microscopy and the use of a scoring system (**Figure 2A**). Whereas the expression of UHMK1 was low to absent in normal pancreatic tissue, its expression was increased corresponding to malignancy as shown by representative images and a diagram (**Figure 2B**). Together, UHMK1 expression positively correlated with the clinical stage because the expression was lower in stage I, higher in stage II, and low to absent in nonmalignant pancreatic tissues. Unfortunately, we were unable to calculate the significance of advanced stages III/IV because only 6 tissues for these stages were available.

### UHMK1 Is an Independent Marker for Overall Survival

**Figure 3:**
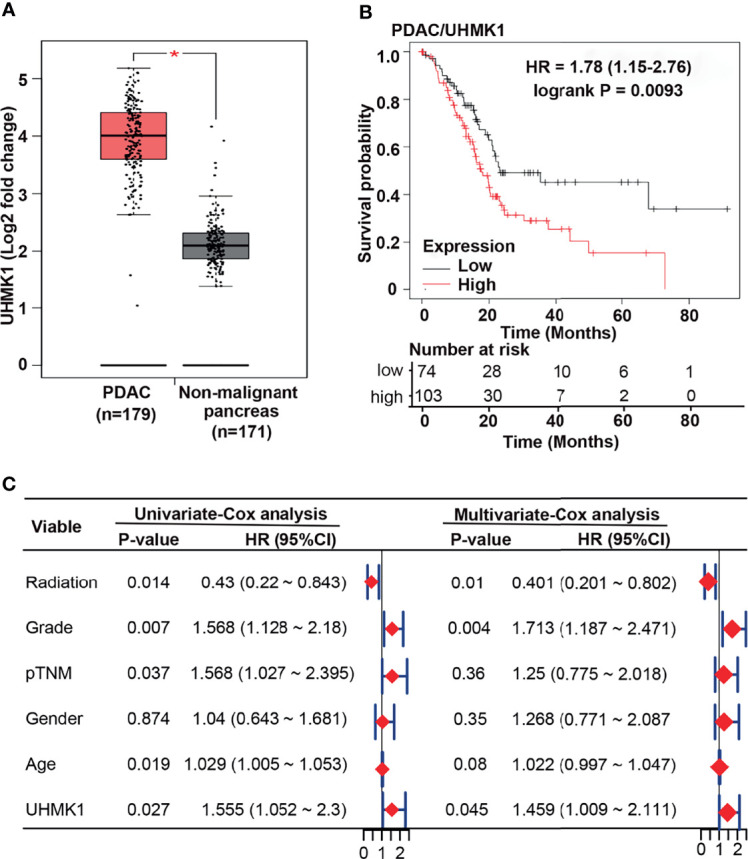
UHMK1 expression correlates with the survival of PDAC patients. **(A)** Using the GEPIA online database, available expression data of UHMK1 in human PDAC (n = 179) and normal pancreatic (n = 171) tissues were identified, and the expression levels with the means ± SD are shown in the diagram. *P < 0.05. Red columns represent PDAC tissue, and gray columns represent tissue from nonmalignant pancreatic tissues. **(B)** Kaplan-Meier analysis of UHMK1 in PDAC. The best cutoff was defined as followed: All possible cutoff values between the lower and higher quartiles are computed, and the best performing threshold is used as a cutoff. The cutoff value was 2390, and the expression range of the probe was 331–5779. The patient data were split according to the cutoff values <2390 = low UHMK1 expression (black line), including 74 patients, and >2390 = high UHMK1 expression (red line), including 103 patients. The Y-axis shows the survival probability, which is the proportion of units that survive beyond a specified time, which is given by the X-axis (Time/Months). The hazard ratio (HR) of 1.78 indicates a 1.78× higher risk of death for patients in the high UHMK1 expression group. The number at risk indicates the number of survivors at the corresponding time point. **(C)** PDAC patients’ clinical data were downloaded from TCGA database, and univariate and multivariate Cox regression analyses were performed in the R studio environment. The risk of death in PDAC patients is expressed as the hazard ratio (HR) according to application of radiation treatment, the tumor grade, the pathologically evaluated tumor/node/metastasis status (pTNM), sex, age, and the level of UHMK1 expression. HR=1 indicates lack of association. HR > 1 indicates an increased risk, and HR < 1 indicates a lower risk. The HR is represented by red diamonds on a scale from 0 to 2.

To examine the impact of UHMK1 in PDAC, we screened TCGA and GTX online databases based on GEPIA for the presence of UHMK1 expression data in PDAC tissues. We found a significant upregulation of UHMK1 in PDAC tissues (n=179) compared to nonmalignant pancreatic tissues (n=171) (**Figure 3A**). These data were confirmed by extracting data from the GEO online database, demonstrating that UHMK1 was more highly expressed in PDAC tissues (n=63, n=24, and n=36) than in nonmalignant paracancerous tissues (n=36) (**Supplementary Figure S4A**). Similarly, *in silico* analysis using the GEPIA database revealed significant UHMK1 upregulation in malignant tissues of other tumor entities compared to adjacent nonmalignant tissues, including breast invasive carcinoma, cervical squamous cell carcinoma and endocervical adenocarcinoma, lymphoid neoplasm diffuse large B-cell lymphoma, esophageal carcinoma, skin cutaneous melanoma, stomach adenocarcinoma, and thymoma (**Supplementary Figure S4B**). The correlation between UHMK1 expression and overall survival was then evaluated by the Kaplan-Meier plotter online database. We divided the available mRNA expression data into high and low UHMK1 expression groups according to the best cutoff value and found a significant association between high UHMK1 expression and shorter overall survival of PDAC patients (**Figure 3B**). To investigate whether UHMK1 expression is an independent risk factor and appropriate for predicting the prognosis of PDAC, a statistical survival model was developed. By performing univariate Cox regression analysis, we examined survival with respect to a single variable and found that UHMK1 expression (P=0.027), age (P=0.019), grade (P=0.007), pTNM stage (P=0.037), and radiation therapy (P=0.014) significantly and independently predicted the overall survival of PDAC patients (**Figure 3C**). Because one variable influences the other, we investigated survival with respect to all identified variables simultaneously by multivariate Cox analysis, which identified UHMK1 (P=0.045), grade (P=0.004), and radiation therapy (P=0.01) as significant risk factors for the prediction of overall survival in PDAC. These data suggested that UHMK1 expression is an appropriate parameter for the prediction of the prognosis of PDAC patients.

### UHMK1 Is Associated With Cancer-Related Pathways

**Figure 4:**
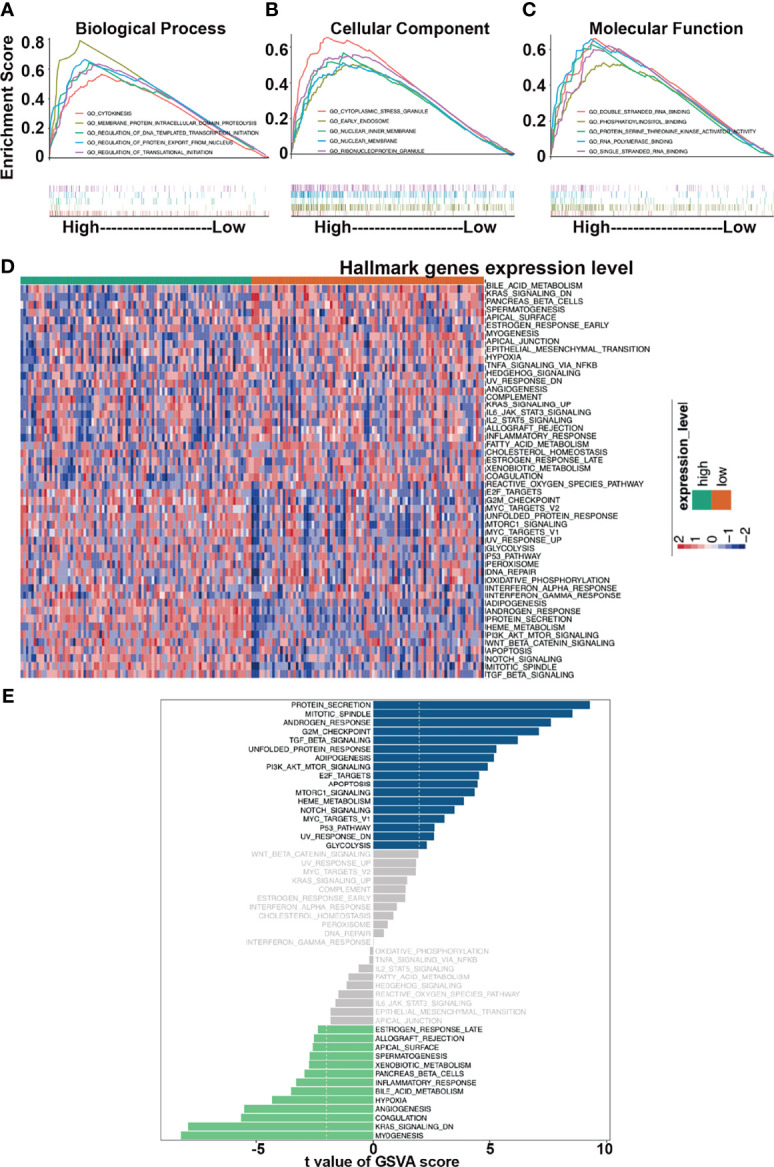
Functional analysis of UHMK1. GO analysis was performed in GSEA software. Top five items are shown. The number of permutations was set to 1000. The top 5 items are shown for each analysis based on the normalized enrichment score (NES). **(A)** Biological process. **(B)** Cellular component. **(C)** Molecular function. **(D)** Hallmark gene sets, which represent specific well-defined biological states or process, were chosen as related gene sets. The R package “GSVA” was utilized to perform GSVA analysis. A heatmap which presents the expression levels of Hallmark pathways relative to high and low expression of UHMK1 is shown. Red: high UHMK1 expression. Blue: low UHMK1expression within a scale from 2 to -2 as indicated. **(E)** mRNA expression of 177 samples was extracted from the TCGA-PAAD database. According to the median of the UHMK1 expression, two groups of high and low UHMK1 expression were split and uploaded to the R-Studio environment and examined by GSVA analysis and the use of the R package “limma”. The threshold was set to t value <2. Samples with high UHMK1 had some gene pathways upregulated (blue bars)/downregulated (green bars).

To highlight the biological function of UHMK1 expression, we analyzed PDAC samples with adjacent information from 19,590 genes, which were selected from TCGA-PAAD database using the TCGAbiolinks R package. Using the median UHMK1 expression in PDAC tissue as the threshold, data were divided into a group with high UHMK1 expression (n=88) and a group with low UHMK1 expression (n=89). These data were evaluated by gene ontology (GO) analysis based on GSEA and the databases “biological processes”, “cellular components”, and “molecular functions” were chosen respectively and the resulting top 5 items are shown according to NES. Regarding the dataset “biological processes”, cytokinesis, membrane protein intracellular domain proteolysis, regulation of DNA templated transcription initiation, regulation of protein export from nucleus, and regulation of translational initiation were found to be enriched (**Figure 4A** and **Table S3**). As for the dataset “cellular components”, cytoplasmic stress granule, early endosome, nuclear inner membrane, nuclear membrane and ribonucleoprotein granule were enriched (**Figure 4B** and **Table S3**). In terms of molecular functions, the double stranded RNA binding, phosphatidylinositol binding, protein serine threonine kinase activator activity, RNA polymerase binding and single stranded RNA binding were enriched (**Figure 4C** and **Table S3**).

To further highlight the functional impact of high UHMK1 expression in PDAC, we performed GSVA analysis. A heatmap was created according to high and low expression of UHMK1. The heatmap was illustrated the enrichment level of each samples in hallmark pathways. Red represented high enrichment score, blue represented low enrichment score (**Figure 4D**). Finally, the differential gene expression between these two groups was evaluated by GSVA and the limma R package, and the results are presented as a bar plot (**Figure 4E**). Significant changes in cancer-related pathways occurred, and alterations were found in protein secretion, cell cycle progression, and signaling pathways, including the TGF-β, PI3K/AKT/mTOR, apoptosis, mTORC1, Notch, Myc, and p53 pathways.

### Knockdown of UHMK1 Suppresses Viability, Migration, and Colony Formation

**Figure 5:**
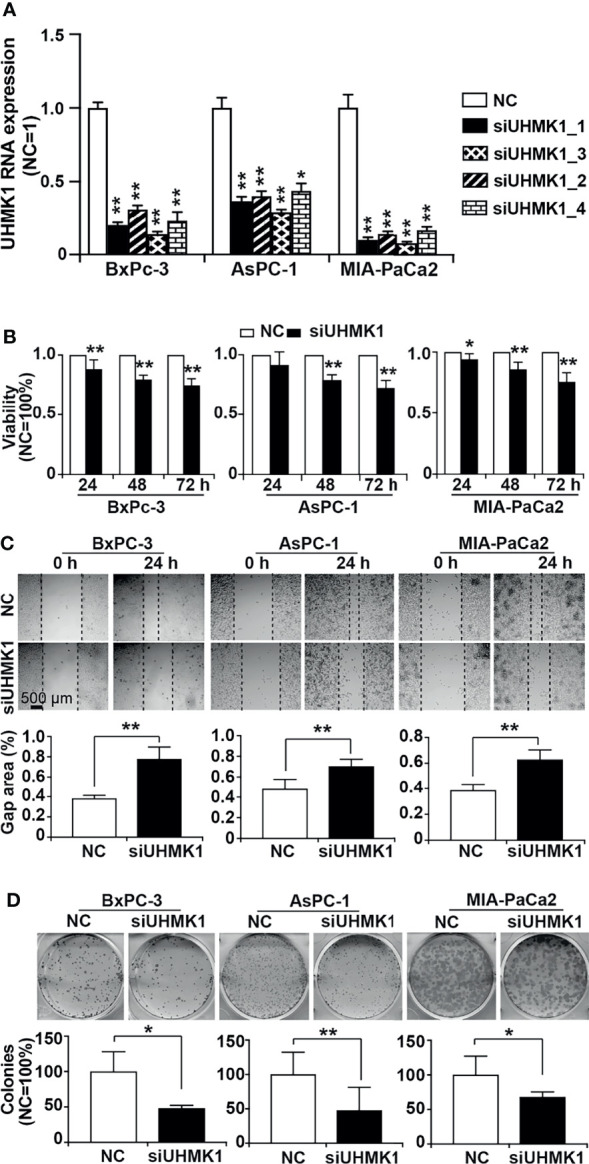
Knockdown of UHMK1 suppresses tumor progression features. **(A)** Four different siRNA constructs of UHMK1 (siUHMK_1, siUHMK1_2, siUHMK1_3, and siUHMK1_4) along with a nonsense siRNA control (NC) were transfected into BxPc-3, AsPC-1, and MIA-PaCa2 cells. RNA was harvested 24 h later, and the expression of UHMK1 was detected by RT-qPCR. The UHMK1 expression levels were normalized to the β-actin housekeeping gene. The fold change of UHMK1 expression was normalized to that in the NC control group, which was set to 1. **(B)** BxPc-3, AsPC-1, and MIA-PaCa2 cells were transfected with siUHMK1 or a nonsense siRNA control (NC). Cell viability was detected by MTT assay at 24 h, 48 h, and 72 h after transfection. The NC control was set to 1. **(C)** Would healing assay. The closure of the wounded region was examined by microscopy 24 h after scratching. Representative images are shown, and the dotted line indicates the gap. The percentage of the gap area was evaluated by ImageJ, and the mean width ± SD is shown in the diagrams. **(D)** Similarly, 24 h after transfection, cells were seeded into 6-well plates at a low density of 400 cells/well and cultured in regular cell culture medium for 14 days. After washing with PBS and fixing with 4% PFA, cells were stained with 0.05% Coomassie blue, and representative images are shown. The number of colonies consisting of at least 50 cells per plate was counted, and the means ± SD are shown in the diagrams. The NC controls were set to 100%. *P < 0.05 and **P < 0.01.

To evaluate the effect of UHMK1 on PDAC progression, we lipotransfected BxPc-3, AsPC-1, and MIA-PaCa2 cells with four different UHMK1 siRNA constructs and evaluated the expression of UHMK1 by RT-qPCR 24 h later. The expression of UHMK1 was strongly inhibited by all siRNAs in all PDAC cells compared to the siRNA control (**Figure 5A**). Among all siRNA constructs, siUHMK1-1 had the strongest and most significant inhibitory effect. Therefore, siUHMK1-1 was used for all subsequent experiments. To assess the impact of UHMK1 on cell viability, transfection of siUHMK1-1 and NC was followed by a MTT assay at 24, 48, and 72 h. The knockdown of UHMK1 significantly inhibited cell viability at all time points, but the effect was time-dependent and most pronounced at 72 h, the percentage ± standard deviation was calculated (BxPc-3: 74.8% ± 0.05; AsPC-1: 72.9% ± 0.06; MIA-PaCa2: 75.8% ± 0.07) (**Figure 5B**). Similarly, we evaluated the effect of UHMK1 on stem cell progression features by scratch and colony-forming assays. Upon UHMK1 knockdown and inhibition of proliferation by serum starvation, we found a significantly slower closure of the wounded region (**Figure 5C**), and UHMK1-deficient cells formed significantly fewer colonies (**Figure 5D**). Above all, MTT assay suggested UHMK1 affected cell viability, wound healing assay gave us a hint that UHMK1 involved in cell migration and colony forming assay indicated that UHMK1 influenced the capacity of colony forming. These results suggested that UHMK1 drives PDAC progression.

### Downregulation of H19 or UHMK1 Inhibits Tumor Growth

**Figure 6:**
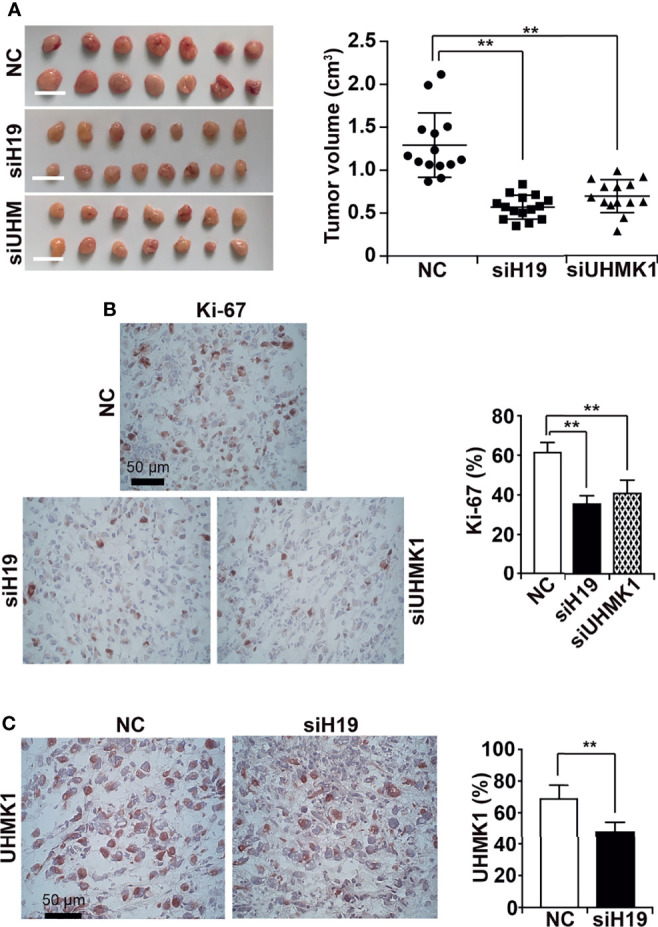
Knockdown of H19 or UHMK1 inhibits tumor growth *in vivo.* **(A)** siH19, siUHMK1, and a nonsense siRNA control (NC) were transfected into MIA-PaCa2 cells. After 24 h, 10^6^ cells of each group were transplanted onto the CAM of each egg (n = 15 eggs/group) on developmental day 9 of the chick embryo. Tumor xenografts were resected on day 18 of chick development, and representative images are shown on the left. The individual xenograft volumes and the mean volumes of each group are presented on the right. **(B)** The expression of the Ki-67 proliferation marker was detected by immunohistochemistry in frozen xenograft sections. Representative images using 400× magnification are shown, and the scale bar represents 50 µm. The percentage of Ki-67-positive cells was quantified by counting the dark red-stained cells in 10 randomly chosen vision fields of each tissue by two independent examiners who were blinded by the conditions. **(C)** The expression of UHMK1 was detected and the percentage of the positive UHMK1 signal was quantified. **P < 0.01.

To evaluate the effect of UHMK1 on tumor growth, we lipotransfected MIA-PaCa2 cells with siH19, siUHMK1, or a control siRNA construct followed by xenotransplantation to the CAM of fertilized chicken eggs at day 9 of chick development. Tumors were resected at day 18, and the tumor volume was determined. Compared to the control, depletion of H19 or UHMK1 rendered smaller tumors in the resected xenografts (**Figure 6A**). Immunohistochemical staining of xenograft sections with Ki-67 demonstrated that proliferation was significantly inhibited by siH19 or siUHMK1 as evaluated by counting the percentage of positively stained cells, as shown in a diagram along with representative staining (**Figure 6B**). To verify the correlation of H19 and UHMK1 expression *in vivo*, we detected UHMK1 expression by immunohistochemistry in H19-deficient xenografts and found a significant downregulation of UHMK1 (**Figure 6C**). To exclude that siH19 or siUHMK1 might have reduced the tumor size by interference with chick development, we confirmed that the weight of each individual chick was not altered between the groups and that liver necrosis did not occur (**Supplementary Figures S5A, B**).

### UHMK1 Expression Is an Appropriate Nomogram Prediction Factor

**Figure 7:**
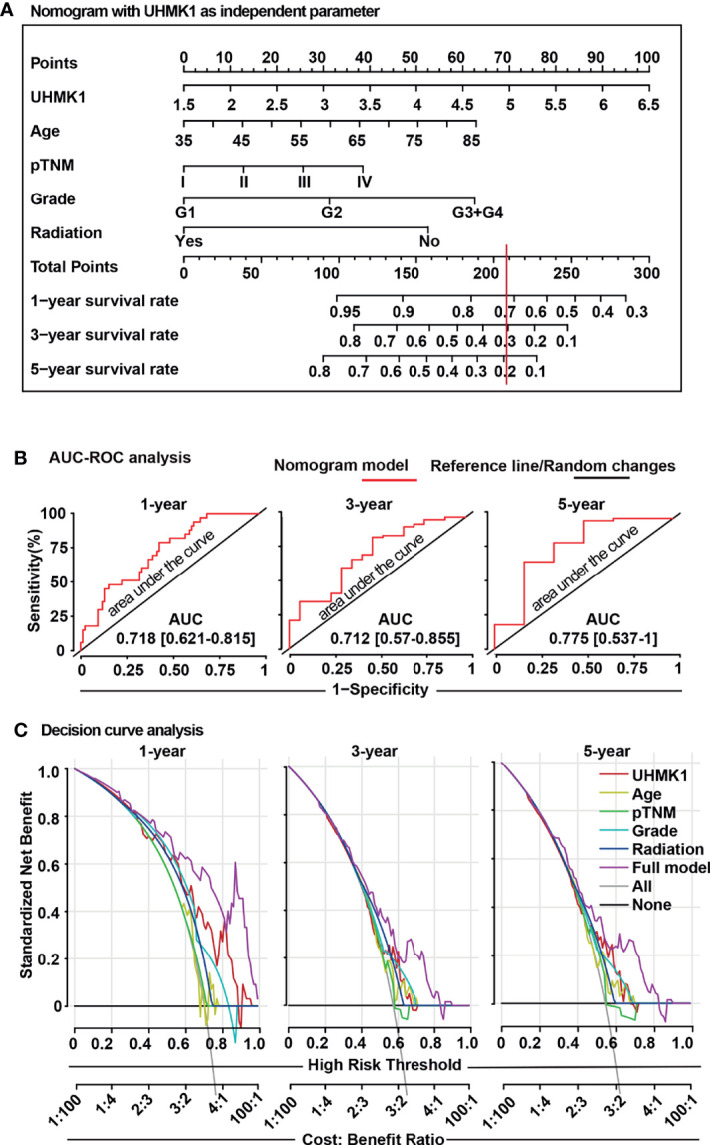
The UHMK1 nomogram reliably predicts the survival probability of PDAC patients. **(A)** A nomogram was constructed using the “RMS package” in R studio software with specific codes. UHMK1 expression data from PDAC patients (n =129) with available clinicopathological parameters were downloaded from TCGA database. Based on univariate Cox regression analysis, a nomogram was established for the prediction of 1-, 3-, and 5-year overall survival. The single parameters of this nomogram were level of UHMK1 expression on a scale from 1.5 to 6.5, age on a scale from 35 to 85, pathologically evaluated tumor/node/metastasis status (pTNM) based on stages I to IV, grade based on G1 to G4 stages, and application of radiation. To estimate the survival probability, the points for each variable was read and summed. A straight line from the sum of all points on the total points axis was then drawn to the 1-, 3-, and 5-year survival axes to determine the respective survival rate. For example, a 60-year-old patient (31 points), who had UHMK1 expression level 5 (70 points), G2 tumor grade (31 points), and TNM stage III (25 points) but who did not undergo radiation therapy (51 points), received a total of 208 points. By drawing a vertical line (red) passing the total points scale at 208, the corresponding 1-, 3-, and 5-year overall survival rates for this patient are 72%, 30%, and 19%, respectively. **(B)** To confirm the predictive power of the nomogram, an “area under the curve” (ROC-AUC) analysis was performed using the “survivalROC”, “survival”, and “riskRegression” R packages in R studio software. The red line represents the nomogram model, and the black line is the reference line for random changes. **(C)** A “decision curve analysis” (DCA) was established to compare the clinical benefits of the nomogram. The “rmda” R package was used in R studio software with specific codes. The gray line represents the treat-all-patients scheme (All), and the black line represents the treat-none scheme (None). The single parameters of UHMK1 expression, age, pTNM, grading, radiation therapy, or all together (full model) were evaluated, and the results are presented in the diagram. The Y-axis represents the standardized net benefit, which is a positive constant quantifying the expected benefit of intervention for a case. The X-axis represents the high-risk threshold, which summarizes the costs and benefits of intervention. The cost:benefit ratio is a ratio of standardized net benefit with or without intervention.

Because nomograms are widely used in oncology to predict personalized prognosis and treatment (21, 22), we added the obtained UHMK1 expression data to a nomogram, containing the confirmed prognostic factors of “age”, “tumor grade”, and “radiation therapy” (**Figure 7A**), which were obtained from TCGA online database. The nomogram was constructed using the “RMS package” in the R studio environment. Scoring points were assigned to each parameter on the individual point scale axes. A total score was calculated by adding the individual points, then projecting the total points to the lower total points scale, which enabled prediction of the 1-, 3-, and 5-year overall survival rates. The allocation of scoring points per individual parameter in the nomogram model is shown in **Supplemental Table S4**. To control how accurately the nomogram predicts survival, we measured the AUC-ROC performance using R language as previously described (23). The AUC values predicted by our nomogram for the 1-, 3-, and 5-year overall survival rates were 0.718, 0.712, and 0.775, respectively (**Figure 7B**), suggesting that our nomogram is an acceptable prediction model (21, 22). Finally, we performed a DCA control experiment to identify the range of threshold probabilities in which the nomogram is of value, the magnitude of benefit, and if the nomogram is worth using in general (24). By DCA, we found that the standardized net benefit of the nomogram model (purple line) was higher than each single item, which gave us a hint that the constructed nomogram had superior clinical utility for PDAC patients (**Figure 7C**). These data indicated that the use of the constructed nomogram to predict prognosis is of greater benefit than using each individual parameter alone.

## Discussion

Here, we demonstrated that the UHMK1 nuclear kinase is induced by lncRNA H19 and showed that both UHMK1 and H19 are strongly involved in PDAC progression. We inhibited H19 expression and performed gene array and bioinformatics analysis along with functional experiments to verify UHMK1 as the top candidate of downregulated genes, closely followed by SGPL1, MIGA1, and SERPINB9. We further investigated UHMK1 expression in PDAC tissues and nonmalignant pancreatic tissues, and we correlated the results to associated clinicopathological data. Elevated UHMK1 levels correlated with advanced TNM stages and predicted the overall survival rate. We established a nomogram with the risk factor of UHMK1 expression and showed that UHMK1 is a reliable parameter and independent predictor of the overall survival of PDAC patients.

We focused on studying UHMK1 because it was at the top of the H19-downregulated candidate genes and because its function in PDAC was previously unknown. To define the role of UHMK1, we assessed the mRNA and protein levels of UHMK1 in PDAC and other tumor entities as well as in nonmalignant pancreatic tissues. We found low UHMK1 expression in nonmalignant pancreatic cells and tissues. In contrast, UHMK1 expression was remarkably increased in several PDAC cell lines and tissues. Furthermore, we detected a significant upregulation of UHMK1 expression in advanced breast cancer, cervical squamous cell carcinoma and endocervical adenocarcinoma, lymphoid neoplasm, diffuse large B-cell lymphoma, esophageal carcinoma, skin cutaneous melanoma, stomach adenocarcinoma, and thymoma. Our results are consistent with previous work, demonstrating enhanced UHMK1 expression in clinical PDAC specimens, PDAC cell lines (25), gastric cancer (19), liver cancer cells (18), and leukemia cells (26).

Our findings imply that depletion of UHMK1 expression inhibited cell viability by performing MTT assay. Meanwhile, we performed wound healing and colony forming assays to confirm our result. *In vivo*, by staining proliferation marker Ki-67 of xenograft tissue which were resected from CAM, we confirmed that the knockdown of UHMK1 expression suppressed the Ki-67 and thereby proliferation. By GSVA, we demonstrated that high expression of UHMK1 correlated with the enrichment of signaling pathways involved in the regulation of the cell cycle, including TGF-β PI3K-AKT-mTOR, Notch, p53 and others. Although the current knowledge about the cellular function of UHMK1 is limited, it is known that UHMK1 signaling is associated with DNA replication, spliceosome biology, and cell cycle regulation (18). A previous study has used FACS sorting to generate UHMK1-deficient cells, which have a reduced number of cells in the S phase and G2/M phases of mitosis (18). These data are in line with another report, confirming that UHMK1 expression leads to cell cycle progression (26). However, there are also contradictory data indicating that UHMK1 silencing does not affect cell cycle progression in U937 leukemia cells (27). The probable reason might be the different microenvironment between solid tumor and non-solid tumor. Finally, one report has indicated that COX5B regulates tumor growth by modulating the AMPK-UHMK1-ERK signaling cascade in hepatoma (28). Interestingly, UHMK1 promotes the progression of gastric cancer through reprogramming of nucleotide metabolism (19). Additionally, YAP-dependent induction of UHMK1 has been reported to support the nuclear enrichment of the MYBL2 oncogene, leading to the proliferation of hepatocellular carcinoma cells as demonstrated in YAP-deficient mice and human hepatocellular carcinoma tissues (18). UHMK1 has been detected as an autoantibody biomarker for serous ovarian cancer using an ELISA platform against a total of 153 serum samples (63 cases with 30 benign disease controls and 60 healthy controls) (20).

Therefore, the signaling pathways underlying the observed UHMK1-regulated progression of PDAC are quite complex and involve the regulation of UHMK1 by lncRNA H19 according to our findings. However, H19 is not the only lncRNA involved in UHMK1 regulation because Xu et al., 2021 stated that lncRNA EBLN3P regulates UHMK1 expression by sponging miR323a-3p, thereby promoting colorectal cancer progression (29). Although it is unknown whether the latter described mechanism is also involved in PDAC progression, our data were consistent with the notion that reduced expression of H19 inhibits PDAC metastasis, which involves the H19-mediated regulation of miR-194 and let-7 (6, 10, 30). Upon siRNA-mediated inhibition of H19 expression, we observed reduced cell viability, migration, invasion, and tumor growth as shown here and in our previous study (12). H19 may exert these effects by inducing several downstream genes. For example, the APOBEC3G tumor promoter is preferentially induced upon induction of H19 expression by the sulforaphane bioactive agent (12). In addition to UHMK1, we detected other strong H19-induced candidate genes, and the H19-mediated upregulation of SGPL1, MIGA1, and SERPINB9 may work together with UHMK1 as we detected a strong correlation of high expression of all of these genes in PDAC tissue but not in nonmalignant pancreatic tissue.

To confirm our results, we performed *in vivo* experiment using fertilized chicken eggs for tumor xenotransplantation. We understand that the mouse model is most common used in researches. However, our former studies illustrated that the pancreatic xenografts can grow fast in fertilized chicken eggs, and the morphology and the expression patterns, progression markers and PDAC markers were comparable with primary patient tissues and their xenograft copies (31). In addition, the tumor environment in chicken egg xenografts were similar compared to primary patient tumors and mouse xenografts (32). Furthermore, we would like to mention that both the subcutaneous xenografts in mouse model and chicken egg xenografts model have their limitations, and cannot entire reflect the pro-fibrotic nature, immunosuppressive tumor microenvironment of PDAC (33). Our constructed chick egg model was several used to successfully established tumor xenotransplantation to the CAM for measuring the tumor growth (12, 34–36). A major advantage of the chicken egg model is that it has natural immunodeficiency. At days 8-9 of chick embryonal development, the blood vessel network is dense enough to bear the growth of the tumor xenograft. On day 18, we resected the xenografts for the reason that the chick hatches on day 21. Therefore, the fertilized chicken eggs are well suited for the short-term studies. By the use of this model, we confirmed that knockdown of UHMK1 and H19 significantly inhibited PDAC xenograft growth but did not completely inhibit it. Likewise, immunohistochemistry confirmed that UHMK1 and Ki-67 expression in xenograft tissue were significantly but not completely reduced following siRNA-mediated knockdown of UHMK1 and H19. To ensure that the siRNA-mediated knockdown was lasting for several days, we transfected the siRNA constructs immediately prior to xenotransplanation. One may speculate that a complete knockout of UHMK1 or H19, e.g. by the use of CRISPR/Cas would have resulted in an even more pronounced inhibition of tumor xenograft growth.

By constructing a statistical survival model, we correlated high UHMK1 expression with a worse prognosis and shorter survival of PDAC patients compared to the parameters of pTNM stage, grading, age, and application of radiation therapy. Unfortunately, we could not compare UHMK1 expression to the effects of the standard chemotherapy regimens, gemcitabine, nab-paclitaxel, or FOLFIRINOX, because a significant number of patient data were not available in online databases.

Finally, we combined UHMK1 expression and clinical parameters to establish a novel prognostic nomogram that individually predicts the 1-, 3-, and 5-year overall survival rates of PDAC patients. To illustrate the reliability of our nomogram, we compared our AUC value to a PDAC nomogram that was constructed by Liu et al., 2021 (37), who reported AUC values of 0.713, 0.753, and 0.823 for 1-, 3-, and 5-year survival, respectively. Liu’s results suggested their constructed nomogram model had the superior predictive power with AUC value more than 0.7. Correspondingly, the AUC values in our nomogram model were 0.718, 0.712, and 0.775 for 1-, 3-, and 5-year survival, respectively. These data demonstrated the satisfactory prediction accuracy of our nomogram as well. According to the AUC and the clinical net benefit control experiments, our nomogram illustrated a better prediction of overall survival compared to the generally accepted prediction parameters of age, pTNM stage, grading, and application of radiation therapy. Nonetheless, before our nomogram can be used clinically, further validation by multicenter, large-scale clinical trials is necessary. Because we calculated online results from different online databases, it may have led to a batch effect, indicating that systematic technical differences can occur when samples are processed and measured in different batches (38).

In conclusion, we characterized UHMK1 as a new lncRNA H19-induced gene and highlighted the function of UHMK1 as a novel progression and prognostic marker and therapeutic target in PDAC. Moreover, the novel UHMK1-based nomogram model provides a more convenient and accurate prediction of the overall survival rate of PDAC patients.

## Materials and Methods

### Tumor Cell Lines

The established human PDAC cell lines, MIA-PaCa2 (RRID : CVCL_0428), BxPc-3 (RRID : CVCL_0186), PANC-1 (RRID : CVCL_0480), and AsPC-1 (RRID : CVCL_0152), as well as the nonmalignant pancreatic ductal cell line, CRL-4023 (RRID : CVCL_C466), were obtained from the American Type Culture Collection (ATCC, Manassas, VA, USA). Gemcitabine-resistant BxGEM cells were selected from parental BxPc-3 cells (RRID : CVCL_0186), as described (39). PDAC cells were cultured at 37°C in high glucose DMEM (Sigma, Deisenhoffen, Germany), 10% FBS (Sigma), and 25 mmol/L HEPES (Thermo Fisher, Dreieich, Germany). CRL-4023 cells were cultured in 75% DMEM without glucose, 2 mM L-glutamine, 1.5 g/L sodium bicarbonate, and 25% M3 Base medium (Incell Corporation LLC, San Antonio, TX, USA). Mycoplasma-negative cultures were ensured monthly by PlasmoTest™ (*In vivo*Gen, San Diego, CA, USA). All cell lines have been authenticated by SNP profiling (Multiplexion, Heidelberg, Germany).

### Patient Tissues

Malignant pancreatic tissues from anonymous patients (n=20) and nonmalignant pancreatic tissues from anonymous brain-dead donors (n=20) were provided by the tissue bank of the European Pancreatic Cancer Center Heidelberg. According to the World Health Organization (WHO), conventional clinical and histological criteria established the clinical diagnoses (**Supplementary Table S1**).

### SiRNA Transfection

PDAC cells were seeded at a concentration of 2×10^5^/well in 6-well plates and cultured in serum-reduced OptiMEM^®^ for 12 hours. AllStars Negative Control siRNA, FlexiTube siRNA siH19-1, FlexiTube siRNA siH19-2, and FlexiTube siRNA directed against human UHMK1 (QIAGEN, Hilden, Germany) were transfected at a concentration of 50 nM using Lipofectamine 2000 (Thermo Fisher Scientific, Dreieich, Germany) according to the manufacturer’s instructions. After 4 h of transfection, the supernatant was discarded, and regular cell culture medium was added. The cells were used for experiments after 24, 48 or 72 h of incubation.

### mRNA Microarray Profiling

MIA-PaCa2 cells were lipotransfected with a nonsense siRNA control or specific siH19-1 and siH19-2 siRNAs (QIAGEN, Hilden, Germany) as described above. The RNeasy Kit (QIAGEN, Hilden, Germany) was used to isolate mRNA. Microarray analysis was performed at the Microarray-Analytic Center of the Medical Faculty Mannheim using the Clariom™ D Assay (Thermo Fisher Scientific, Dreieich, Germany). Heatmaps and volcano plots were created with R Studio (https://rstudio.com/products/rstudio/). The “limma” R package was applied to normalize the data and to identify differentially expressed genes between control cells and siH19-1 or siH19-2-transfected cells. Genes with a fold change >1 and a P value <0.05 were considered significantly differentially regulated genes. The results were prepared as heatmaps or volcano plots using the “ggplot2” R package.

### mRNA Extraction and RT-qPCR

The RNeasy Mini Kit (QIAGEN, Hilden, Germany) was used to isolate mRNA. For reverse transcription, the High-Capacity RNA-to-DNA™ Kit (Thermo Fisher Scientific, Dreieich, Germany) was utilized according to the manufacturer’s instructions. DNA was amplified using PowerUp™ SYBR™ Green Master Mix (Thermo Fisher Scientific, Germany) by RT-qPCR. The following primer sequences were used: UHMK1 forward, 5´-AGAGAAACCATGGGCAGAAG-3´; UHMK1 reverse, 5´-CAAGCCATGAAACAGCATCT-3´; β-actin forward, 5´-AATCGTGCGTGACATTAAGGAG-3´; and β-actin reverse, 5´-ACTGTGTTGGCGTACAGGTCTT-3. The concentration of each primer was 500 nM. The gene expression levels were normalized to the β-actin housekeeping gene. The qPCR conditions were as follows: 40 cycles of denaturation at 95°C for 15 sec, annealing at 56°C for 15 sec, and extension at 72°C for 1 min. The results are presented as the relative expression value, which was calculated by the 2^-ΔΔCt^ method (40).

### Western Blot Analysis

After treatment, cells were lysed with RIPA lysis buffer (Abcam, Cambridge, UK), and total protein was purified by a standard protocol. Protein concentration was determined by the BCA Protein Assay Kit (Abcam, Cambridge, UK). Before SDS-PAGE separation, the samples were denatured by boiling for 5 min and then kept on ice. The separated proteins were transferred from the gel to a PVDF membrane by a semidry system. The membrane was blocked by incubation in 3% BSA solution, incubated with primary antibodies, washed, and incubated with IRDye® infrared dye-conjugated secondary antibodies (LI-COR Biosciences, Bad Homburg, Germany). The infrared intensity was measured with an Odyssey CLx Infrared Imaging System (LI-COR). Rabbit polyclonal antibodies against UHMK1 (PA550622, Invitrogen, Germany), GAPDH (Cell Signaling Technology, Danvers, MA, USA), and IRDye^®^ 800CW goat anti-rabbit IgG secondary antibody (LI-COR) were used.

### Immunohistochemical Staining

Immunohistochemistry on 6-µm frozen or paraffin-embedded tissue sections was performed as previously described (41). Primary antibodies included rabbit polyclonal antibodies against UHMK1 (PA550622, Invitrogen, Germany) and Ki67 (Abcam, Cambridge, UK). Goat anti-rabbit biotinylated IgG (Vector, Burlingame, CA, USA) was the secondary antibody. ImageJ was used to calculate the intensity of the signal emitted from positively stained cells.

### Gene Expression Profiling Interactive Analysis (GEPIA)

GEPIA is an online database (http://gepia.cancer-pku.cn) that contains tumor data from The Cancer Genome Atlas (TCGA, https://www.cancer.gov/about-nci/organization/ccg/research/structural-genomics/tcga) and the Genotype-Tissue Expression (GTEx, https://gtexportal.org/home/) databases. GEPIA was used to analyze the expression of UHMK1 in tumor and normal tissue derived from PDAC and other tumor entities.

### Tumor IMmune Estimation Resource 2.0 (TIMER 2.0)

TIMER (http://timer.cistrome.org) is a comprehensive resource for systematical analysis in diverse cancer types to explore immune association, cancer exploration, and immune estimation. Within the TIMER database, a Pearson correlation analysis was performed to compare the expression of UHMK1 with SGPL1, SERPINB9, and MIGA1.

### Kaplan-Meier Plotter Survival Analysis

UHMK1 was identified as a survival biomarker by Kaplan-Meier survival analysis and the Kaplan-Meier plotter online database (https://kmplot.com/analysis/), which contains expression data from 54,000 genes with corresponding survival data from 21 different tumor entities, including PDAC. The Kaplan-Meier plotter database includes TCGA, GEO, and EGA. The purpose of this online database is to discover and validate survival biomarkers. The Kaplan-Meier plotter online database was used to detect the overall survival rate of patients with low or high UHMK1 expression in PDAC tissue samples. Auto select best cutoff was chosen in the analysis.

### Cox Regression Analysis

The clinical data of PDAC patients were downloaded from the TCGA database, and univariate and multivariate Cox regression analyses were performed in the R studio environment. The risk of death in PDAC patients is expressed as the hazard ratio (HR) according to application of radiation treatment, the tumor grade, the pathologically evaluated tumor/node/metastasis status (pTNM), sex, age, and the level of UHMK1 expression. HR=1 indicates lack of association. HR>1 indicates an increased risk, and HR<1 indicates a lower risk.

### Gene Set Enrichment Analysis (GSEA) and Gene Set Variation Analysis (GSVA)

GSEA 4.0.3 software was used for gene set enrichment analysis. GSEA is a computational method that determines whether an *a priori* defined set of genes shows statistically significant, concordant differences between two biological states (42). Gene Ontology (GO) analysis was conducted using the GSEA software, which was download from Molecular Signature Database (MSigDB) (https://www.gsea-msigdb.org/gsea/msigdb). The GSVA R package was used for gene set variation analysis (GSVA) in the R Studio environment. The h.all.v7.1.symbols.gmt gene set database (https://www.gsea-msigdb.org/gsea/msigdb) was used for enrichment analyses. For the GSVA, the related gene sets, including c5.bp.v7.1.symbols.gmt, c5.cc.v7.1.symbols.gmt, and c5.mf.v7.1.symbols.gmt, were downloaded from MSigDB (https://www.gsea-msigdb.org/gsea/msigdb).

### Cell Viability Assay

Twenty-four hours after transfecting with siUHMK1, BxPc-3, AsPC-1 and MIA-PACA2 (5×10^4^ cells/well) were seeded into 96-well microplates. After transfection of siUHMK1 at 24, 48, and 72h, 10 µL of 3-(4,5-dimethylthiazol-2-yl)-2,5-diphenyltetrazolium bromide (MTT) was added and incubated for 4 h. Depending on active NADPH-dependent cellular oxidoreductase enzymes, which are present in the functional mitochondria of viable cells, MTT was reduced to insoluble, purple formazan. Subsequently, the medium was carefully discarded, and 200 µL of DMSO was added to dissolve the formazan crystals by shaking on a plate shaker. The color intensity was quantified at a wavelength of 560 nm by spectrophotometry. The viability was evaluated by subtracting the DMSO background, calculating the mean values of each group (n=8), and calculating the standard deviations. The controls of each cell line were set to 100%.

### Wound-Healing Assay

Twenty-four hours after lipotransfection, 5×10^5^ cells/well were seeded in 6-well plates. Upon reaching a confluency of approximately 90%, a scratch was made with the tip of a 10-µL pipette in the middle of the cell layer, and this time point was set as 0 h. Cells were washed twice with PBS, and the width of the gap area was determined. Cells were then cultured in serum-free medium to stop proliferation. After incubation for 24 h and 48 h at 37°C, images were acquired using a Nikon Eclipse TS 100-F inverted microscope. The width of the gap area was measured with ImageJ (https://imagej.net/Downloads).

### Colony-Forming Assay

Twenty-four hours after lipotransfection, 400 cells/well were seeded in 6-well plates and cultured without medium change for 14 days. Cells were then washed with 10 mL of PBS and fixed with 2 mL of 4% paraformaldehyde (PFA) for 10 min. The fixation solution was replaced with 2 mL of 70% EtOH, which was incubated for 10 min. Finally, cells were stained with 0.05% Coomassie Blue, washed with water, and dried overnight. The percentage of colonies was evaluated by normalizing the number of transfected cell colonies to the number of colonies obtained from cells transfected with the negative control siRNA.

### Tumor Xenotransplantation

Fertilized chicken eggs were purchased from a local ecological hatchery (Geflügelzucht Hockenberger, Eppingen, Germany), and the eggs were prepared for transplantation as previously described (43). Before xenotransplantation, MIA-PaCa2 cells were transfected with siH19, siA3G, or negative control siRNA. At day 9 of chick development, 10^6^ transfected MIA-PaCa2 cells/egg were transplanted onto the chorioallantois membrane (CAM) as previously described (43). At day 18 of chick development, the embryos were humanely euthanized by injection of 10 µL of a 25 mg/ml Ketanest^®^ solution (Pfizer Pharma PFE GmbH, Berlin, Germany) into a CAM vessel followed by resection of the tumor xenografts. The tumor volume was measured 3-dimensionally by a USB microscope camera (eScope, Oitez, Hong Kong), and digital image editing was performed using a customized mount.

### Construction of a UHMK1-Based Nomogram

Nomographical two-dimensional alignment charts (nomograms) are widely used for individual prediction of cancer prognosis (21). By using univariate Cox regression analysis, cancer-related variables can be filtered for nomogram construction (44). Based on the univariate Cox regression analysis of UHMK1 expression and clinical data in TCGA database, a nomogram was constructed to predict the 1-, 3-, and 5-year overall survival rates using the “RMS package” in R studio. To further confirm the superiority of the nomogram, the area under the curve (AUC) was implemented to evaluate the accuracy of prognostic prediction of the nomogram, while receiver operating characteristic (45) analysis was used to determine the sensitivity and specificity. Decision curve analysis (DCA) was used to compare the clinical benefits of the constructed nomogram. The “survivalROC”, “survival”, “riskRegression”, and “rmda” R packages were also used.

### Data Extraction From GEO

Three independent datasets, GSE57495, GSE77435, and GSE15471, were downloaded from GEO (http://www.ncbi.nlm.nih.gov/geo/). The GSE57495, GSE77435, and GSE15471 datasets included 63 PDAC tissues, 24 PDAC tissues, and 36 PDAC tissues, respectively, with 36 matched normal paracancerous tissues, and they were analyzed for UHMK1 expression.

### Statistical Analysis

The quantitative data are presented as the mean values and standard deviations from at least three independent experiments. The significance of the data was analyzed with Student’s t test, which was corrected for multiple comparisons with the Bonferroni-Holm method. A Pearson correlation analysis was performed to measure the linear correlation between two variables, including UHMK1 expression vs. SGPL1 expression, SERPIMB9 expression, or MIGA1 expression. JMP software (SAS, Heidelberg, Germany) was used to analyze the gene microarray data. R studio software version 4.0.3 was also used for statistical analyses. P<0.05 was considered statistically significant. In GSEA, a false discovery rate (FDR) of 5% was used to adjust for multiple testing. *P<0.05, **P<0.01, and ***P<0.001.

## Data Availability Statement

The datasets presented in this study can be found in online repositories. The names of the repository/repositories and accession number(s) can be found in the article/**Supplementary Material**.

## Ethics Statement

Patient materials were obtained under the approval of the Ethical Committee of the University of Heidelberg after receiving written informed consent from patients. The diagnoses were established by conventional clinical and histological criteria according to the WHO and the anonymous patient data are provided. All surgical resections were indicated by the principles and practice of oncological therapy. Established tumor cell lines were xenotransplanted to fertilized chicken eggs. Since the chick embryo until day 18 of development is not considered as animal, an animal ethics statements is not required.

## Author Contributions

IH and YL, conception and design. YL and SH, development of methodology. YL, SH, BY, HJ, LZ, and JG, acquisition of data. YL, BY, and IH, analysis and interpretation of data. YL and IH, writing, review, and revision of the manuscript. All authors contributed to the article and approved the submitted version.

## Funding

This study was supported by the China Scholarship Council in the form of scholarships to YL and SH. IH was supported by grants from the German Research Council (DFG HE 3186/15–1), Karsten Burmeister - BIMAG Bau- und Industriemaschinen GmbH, Heidelberger Stiftung Chirurgie, Dietmar Hopp-Stiftung, and Klaus Tschira Stiftung.

## Conflict of Interest

The authors declare that the research was conducted in the absence of any commercial or financial relationships that could be construed as a potential conflict of interest.

## Publisher’s Note

All claims expressed in this article are solely those of the authors and do not necessarily represent those of their affiliated organizations, or those of the publisher, the editors and the reviewers. Any product that may be evaluated in this article, or claim that may be made by its manufacturer, is not guaranteed or endorsed by the publisher.

## Abbreviations

AUC, Area under the curve; CAM, Chorioallantois membrane; DCA, Decision curve analysis; ES, Enrichment score; FDR, False discovery rate; GEPIA, Gene expression profiling interactive analysis; GO, Gene Ontology; GSEA, Gene Set Enrichment Analysis; GSVA, Gene Set Variation Analysis; siH19-1, H19 siRNA No. 1; siH19-2, H19 siRNA No. 2; H&E, Hematoxylin and eosin; LncRNA, Long noncoding RNA; MSigDB, Molecular Signature Database; MTT, 3-(4,5-dimethylthiazol-2-yl)-2,5-diphenyltetrazolium bromide; OS, Overall survival; PDAC, Pancreatic ductal adenocarcinoma; ROC, Receiver operating characteristic; UHMK1, U2AF homology motif kinase 1; NES, Normalized enrichment score.
