# Supplementary material for: UHMK1 Is a Novel Marker for Personalized Prediction of Pancreatic Cancer Prognosis
Source: Front Oncol. 2022 Mar 10;12:834647. doi: 10.3389/fonc.2022.834647 (PMC8960145; doi:10.3389/fonc.2022.834647)
Supplement: Supplementary file 1 [file DataSheet_1.pdf]

Supplemental Information

UHMK1 is a novel marker for personalized prediction of pancreatic cancer prognosis

Yiqiao Luo, Shanshan Han, Bin Yan, Huihui Ji, Jury Gladkich, Ingrid Herr

Table of Content

Supplemental Tables

Table S1 Anonymous patient information for tissues from our clinic 2
Table S2 Clinico-pathological information for tissue of the microarray 3
Table S3 Gene ontology (GO)-analysis of UHMK1 expression 6
Table S4 Nomogram: Scoring points per individual parameter 6

Supplemental Figures with Legends

Figure S1 Selection of six lncRNA H19-induced genes 7
Figure S2 SGPL1, SERPINB9, and MIGA1 expression correlates with UHMK1 expression in PDAC tissue 8
Figure S3 Original, crude Western blot images... 9
Figure S4 UHMK1 expression is upregulated in PDAC and other types of cancer ..10
Figure S5 siH19 or siUHMK1 downregulation is not associated with side effects *in vivo* 11

Supplemental Table S1 Anonymous patient information for tissues from our clinic

Intern No.	Tissue No.	Gender	Age	T	N	M	No. LNs positive	No. LNs examined	G	R	Histology
2913	1	F	64	1	1	0	3	26	2	1	PDAC
6053	2	M	51	3	1	0	7	42	2	1	
6476	3	M	78	2	2	1	30	54	2	1	
6508	4	M	73	2	4	1	11	26	3	1	
6566	5	F	78	3	2	1	6	38	3	1	
6569	6	M	67	3	4	0	13	26	2	1	
6571	7	F	58	3	4	1	8	55	3	1	
6633	8	F	63	3	2	0	16	72	3	1	
6805	9	M	57	2	4	0	13	29	3	1	
6812	10	F	68	1	4	1	11	41	2	0	
6834	11	M	57	2	4	1	9	39	3	1	
6863	12	M	78	2	2	0	18	42	3	1	
6871	13	F	81	3	4	0	15	19	2	1	
6872	14	M	75	3	2	1	9	57	2	1	
6893	15	F	65	2	2	0	22	45	3	1	
7059	16	M	69	3	1	1	3	34	2	1	
7094	17	F	65	2	2	1	5	24	3	1	
7096	18	F	77	2	4	0	11	59	2	0	
7105	19	F	75	2	2	0	5	28	2	1	
7121	20	M	66	3	4	0	17	40	2	1	
1166	1	M	69	Does not apply							Non-malignant pancreas tissue from brain-dead donors
1207	2	F	19								
1220	3	M	38								
1234	4	F	63								
1242	5	M	7								
1244	6	M	58								
1303	7	M	58								
1366	8	M	59								
1395	9	M	38								
1413	10	F	63								
1417	11	NA	58								
1434	12	F	40								
1516	13	F	60								
1517	14	F	40								
1527	15	F	44								
1550	16	F	70								
1590	17	M	70								
1611	18	F	59								
1663	19	M	58								
1673	20	M	52								

No.: Number; **F:** Female; **M:** Male; **PDAC:** Pancreatic ductal adenocarcinoma; **T1:** Tumor limited to the pancreas, 2 cm or less in greatest dimension; **T2:** Tumor limited to the pancreas, more than 2 cm in greatest dimension; **T3:** Tumor extends beyond pancreas; **T4:** Tumor involves coeliac axis or superior mesenteric artery; **LNs:** Lymph-node metastasis; **M0:** No distant metastasis; **M1:** Distant metastasis; **G1:** Well-differentiated; **G2:** Moderately differentiated; **G3:** Poorly differentiated; **R0:** Indicates a microscopically margin-negative resection; **R1:** Indicates the removal of all macroscopic visible tumor tissue, but the microscopic examination of margins reveals that there is tumor tissue. **NA:** Not available.

Table S2 Anonymous clinico-pathological information for tissue of the microarray

Pos	No.	Sex	Age	Organ	Pathology diagnosis	Grade	Stage	TNM	Type
A1	1	F	67	Pancreas	Duct adenocarcinoma	1	III	T3N1bMo	Malignant
A2	2	F	48	Pancreas	Duct adenocarcinoma	1	IIA	T3N0M0	Malignant
A3	3	F	47	Pancreas	Duct adenocarcinoma	1	IIA	T3N0M0	Malignant
A4	4	M	59	Pancreas	Duct adenocarcinoma	1	IIB	T2N1M0	Malignant
A5	5	F	44	Pancreas	Adenocarcinoma (sparse)	-	IIB	T3N1M0	Malignant
A6	6	M	34	Pancreas	Duct adenocarcinoma	1	IIA	T3N0M0	Malignant
A7	7	M	49	Pancreas	Duct adenocarcinoma	1	IB	T2N0M0	Malignant
A8	8	M	56	Pancreas	Duct adenocarcinoma	1	IB	T2N0M0	Malignant
A9	9	M	49	Pancreas	Duct adenocarcinoma	1	IB	T2N0M0	Malignant
A10	10	M	67	Pancreas	Duct adenocarcinoma	1	IB	T2N0M0	Malignant
A11	11	M	48	Pancreas	Duct adenocarcinoma	1	IB	T2N0M0	Malignant
A12	12	F	56	Pancreas	Duct adenocarcinoma	-	IB	T2N0M0	Malignant
B1	13	F	58	Pancreas	Duct adenocarcinoma	1	IIB	T2N1M0	Malignant
B2	14	M	52	Pancreas	Duct adenocarcinoma	1	IIB	T3N1M0	Malignant
B3	15	F	51	Pancreas	Duct adenocarcinoma	-	IIA	T3N0M0	Malignant
B4	16	M	55	Pancreas	Duct adenocarcinoma	1	IIA	T3N0M0	Malignant
B5	17	F	62	Pancreas	Duct adenocarcinoma with necrosis	2	III	T4N0M0	Malignant
B6	18	M	56	Pancreas	Duct adenocarcinoma	2	IB	T2N0M0	Malignant
B7	19	M	60	Pancreas	Duct adenocarcinoma	2	IA	T2N0M0	Malignant
B8	20	M	52	Pancreas	Duct adenocarcinoma	-	IIA	T3N0M0	Malignant
B9	21	M	42	Pancreas	Hyperplasia of duct epithelium	-	IB	T2N0M0	Malignant
B10	22	M	54	Pancreas	Duct adenocarcinoma	-	IIA	T3N0M0	Malignant
B11	23	F	51	Pancreas	Duct adenocarcinoma	1	IIA	T3N0M0	Malignant
B12	24	F	54	Pancreas	Duct adenocarcinoma	2	IB	T2N0M0	Malignant
C1	25	M	54	Pancreas	Duct adenocarcinoma	2	IIA	T3N0M0	Malignant
C2	26	M	39	Pancreas	Duct adenocarcinoma	2	IIA	T3N0M0	Malignant
C3	27	F	41	Pancreas	Duct adenocarcinoma	-	IIA	T3N0M0	Malignant
C4	28	F	68	Pancreas	Duct adenocarcinoma	2	IIA	T3N0M0	Malignant
C5	29	F	44	Pancreas	Duct adenocarcinoma	2	IIB	T3N1M0	Malignant
C6	30	M	42	Pancreas	Duct adenocarcinoma	1	IIA	T3N0M0	Malignant
C7	31	M	53	Pancreas	Duct adenocarcinoma	2	IB	T2N0M0	Malignant
C8	32	M	51	Pancreas	Duct adenocarcinoma	1	IIA	T3N0M0	Malignant
C9	33	M	48	Pancreas	Duct adenocarcinoma	1	IIA	T3N0M0	Malignant
C10	34	M	57	Pancreas	Duct adenocarcinoma	-	IIA	T3N0M0	Malignant
C11	35	F	64	Pancreas	Duct adenocarcinoma	1	IIA	T3N0M0	Malignant
C12	36	M	49	Pancreas	Duct adenocarcinoma	2	IIA	T3N0M0	Malignant
D1	37	M	72	Pancreas	Duct adenocarcinoma	1	IIA	T3N0M0	Malignant
D2	38	F	72	Pancreas	Duct adenocarcinoma	2	IIA	T3N0M0	Malignant
D3	39	M	59	Pancreas	Duct adenocarcinoma	2	IIA	T3N0M0	Malignant
D4	40	F	45	Pancreas	Duct adenocarcinoma	2	III	T3N2M0	Malignant
D5	41	F	60	Pancreas	Duct adenocarcinoma	2	IV	T2N1M1	Malignant
D6	42	M	52	Pancreas	Chronic inflammation of pancreas tissue	-	IA	T1N0M0	Malignant
D7	43	F	44	Pancreas	Duct adenocarcinoma with necrosis	2	IB	T2N0M0	Malignant
D8	44	F	46	Pancreas	Duct adenocarcinoma	2	IB	T2N0M0	Malignant
D9	45	M	52	Pancreas	Duct adenocarcinoma	2	IB	T2N0M0	Malignant
D10	46	M	52	Pancreas	Duct adenocarcinoma	2	IB	T2N0M0	Malignant
D11	47	F	53	Pancreas	Duct adenocarcinoma	2	IIB	T2N1M0	Malignant
D12	48	M	40	Pancreas	Duct adenocarcinoma	1	IIB	T2N1bM0	Malignant
E1	49	M	57	Pancreas	Duct adenocarcinoma	1	IIA	T3N0M0	Malignant
E2	50	M	31	Pancreas	Duct adenocarcinoma	1	IIA	T3N0M0	Malignant
E3	51	M	44	Pancreas	Duct adenocarcinoma	2	IIA	T3N0M0	Malignant
E4	52	M	61	Pancreas	Adenocarcinoma	3	IV	T3N0M1	Malignant
E5	53	M	51	Pancreas	Duct adenocarcinoma	2	IIA	T3N0M0	Malignant
E6	54	M	59	Pancreas	Duct adenocarcinoma	2	IA	T1N0M0	Malignant
E7	55	M	44	Pancreas	Adenocarcinoma	3	IIA	T3N0M0	Malignant
E8	56	M	45	Pancreas	Adenocarcinoma	3	IIB	T2N1M0	Malignant
E9	57	M	41	Pancreas	Duct adenocarcinoma	2	IB	T2N0M0	Malignant
E10	58	F	72	Pancreas	Duct adenocarcinoma	2	IIA	T3N0M0	Malignant
E11	59	F	51	Pancreas	Duct adenocarcinoma	-	IIA	T3N0M0	Malignant
E12	60	F	42	Pancreas	Duct adenocarcinoma	2	IB	T2N0M0	Malignant
F1	61	F	39	Pancreas	Duct adenocarcinoma	2	IIA	T3N0M0	Malignant
F2	62	F	51	Pancreas	Duct adenocarcinoma with necrosis	2	IIA	T3N0M0	Malignant
F3	63	M	62	Pancreas	Duct adenocarcinoma	1	IIA	T3N0M0	Malignant
F4	64	M	60	Pancreas	Duct adenocarcinoma	2	IB	T2N0M0	Malignant
F5	65	F	53	Pancreas	Duct adenocarcinoma	2	IIA	T3N0M0	Malignant
F6	66	M	77	Pancreas	Duct adenocarcinoma	2	IA	T1N0M0	Malignant
F7	67	M	47	Pancreas	Adenocarcinoma	3	IIA	T3N0M0	Malignant
F8	68	M	67	Pancreas	Adenocarcinoma	3	IIA	T3N0M0	Malignant
F9	69	M	78	Pancreas	Adenocarcinoma	3	IB	T2N0M0	Malignant

F10	70	M	62	Pancreas	Adenocarcinoma	3	IIA	T3N0M0	Malignant
F11	71	M	50	Pancreas	Adenocarcinoma	3	IB	T2N0M0	Malignant
F12	72	M	55	Pancreas	Adenocarcinoma	3	IIB	T3N1M0	Malignant
G1	73	F	48	Pancreas	Adenocarcinoma	3	IIA	T3N0M0	Malignant
G2	74	M	50	Pancreas	Adenocarcinoma	3	IIB	T3N1M0	Malignant
G3	75	F	53	Pancreas	Adenocarcinoma with necrosis	3	IB	T2N0M0	Malignant
G4	76	M	50	Pancreas	Adenocarcinoma	3	IB	T2N0M0	Malignant
G5	77	F	23	Pancreas	Adenocarcinoma	3	IB	T2N0M0	Malignant
G6	78	F	56	Pancreas	Adenocarcinoma	-	IIB	T2N1bM0	Malignant
G7	79	M	56	Pancreas	Undifferentiated carcinoma	-	IIA	T3N0M0	Malignant
G8	80	F	54	Pancreas	Mixed acinar-neuroendocrine carcinoma	-	IB	T2N0M0	Malignant
G9	81	M	52	Pancreas	Adenosquamous carcinoma	-	IIA	T3N0M0	Malignant
G10	82	F	49	Pancreas	Adenosquamous carcinoma	-	IIB	T3N1M0	Malignant
G11	83	M	50	Pancreas	Adenosquamous carcinoma	-	IIA	T3N0M0	Malignant
G12	84	M	62	Pancreas	Squamous cell carcinoma	2	IIA	T3N0M0	Malignant
H1	85	F	52	Pancreas	Carcinoid	-	IIA	T3N0M0	Malignant
H2	86	F	50	Pancreas	Carcinoid	-	IIA	T3N0M0	Malignant
H3	87	M	51	Pancreas	Atypical carcinoid	-	III	T4N0M0	Malignant
H4	88	M	42	Pancreas	Neuroendocrine carcinoma	-	IIA	T3N0M0	Malignant
H5	89	M	53	Pancreas	Acinic cell carcinoma	-	IB	T2N0M0	Malignant
H6	90	F	33	Pancreas	Solid pseudo-papillary carcinoma	-	IIA	T3N0M0	Malignant
H7	91	M	42	Pancreas	Solid pseudo-papillary carcinoma	-	IB	T2N0M0	Malignant
H8	92	F	21	Pancreas	Pancreatic tissue	-	-		Normal
H9	93	M	47	Pancreas	Pancreatic tissue	-	-		Normal
H10	94	M	38	Pancreas	Pancreatic tissue	-	-		Normal
H11	95	M	40	Pancreas	Pancreatic tissue	-	-		Normal
H12	96	F	38	Pancreas	Pancreatic tissue	-	-		Normal
-	-	M	42	Adrenal gland	Pheochromocytoma (tissue marker)	-	-		Malignant

Table S3 Gene ontology (GO)-analysis of UHMK1 expression in PDA

	Function	ES	NES	FDR
**Biological Process**	Cytokinesis	0.57	2.11	0.039
	Membrane protein intracellular domain proteolysis	0.79	2.16	0.045
	Regulation of DNA templated transcription initiation	0.64	2.11	0.033
	Regulation of protein export from nucleus	0.67	2.09	0.038
	Regulation of translational initiation	0.63	2.13	0.045
**Cellular Component**	Cytoplasmic stress granule	0.65	2.08	0.023
	Early endosome	0.50	2.02	0.020
	Nuclear inner membrane	0.57	2.08	0.016
	Nuclear membrane	0.51	2.03	0.019
	Ribonucleoprotein granule	0.56	2.10	0.036
**Molecular Function**	Double stranded RNA binding	0.66	2.13	0.021
	Phosphatidylinositol binding	0.53	2.03	0.027
	Protein serine threonine kinase activator activity	0.63	2.02	0.024
	RNA polymerase binding	0.66	2.04	0.033
	Single stranded RNA binding	0.62	2.06	0.035

This table relates to Fig. 4A, B, C

ES: Enrichment score
NES: Normalized enrichment score
FDR: False discovery rate

Table S4 Nomogram: Scoring points per individual parameter

UHMK1		Age		pTNM		Grade		Radiation		Survival Time					
										1-year		3-years		5-years	
**ES**	**N**	**Y**	**N**	**S**	**N**	**G**	**N**	**RT**	**N**	**SR**	**N**	**SR**	**N**	**SR**	**N**
1.5	0	35	0	I	0	G1	0	Yes	0	0.95	99	0.8	110	0.8	90
2	10	40	6	II	13	G2	31	No	53	0.9	141	0.7	137	0.7	118
2.5	20	45	13	III	26	G3/4	63			0.8	185	0.6	159	0.6	139
3	30	50	19	IV	38					0.7	213	0.5	177	0.5	157
3.5	40	55	25							0.6	234	0.4	193	0.4	173
4	50	60	31							0.5	252	0.3	209	0.3	189
4.5	60	65	38							0.4	269	0.2	226	0.2	207
5	70	70	44							0.3	285	0.1	247	0.1	228
5.5	80	75	50												
6	90	80	57												
6.5	100	85	63												

ES: UHMK1 expression score; **N:** Nomogram scoring points; **Y:** Life years**; S:** pTNM stage**; G:** Grade**; RT:** Radiation therapy obtained; **SR:** Survival rate; By constructing the multivariate Cox regression, the regression coefficient of each variable was obtained. By setting the score of the variable with the largest regression coefficient to 100, all other variables are converted accordingly and as described [1].

Reference

[1] A. Iasonos, D. Schrag, G.V. Raj, K.S. Panageas, How to build and interpret a nomogram for cancer prognosis, J Clin Oncol, 26 (2008) 1364-1370.

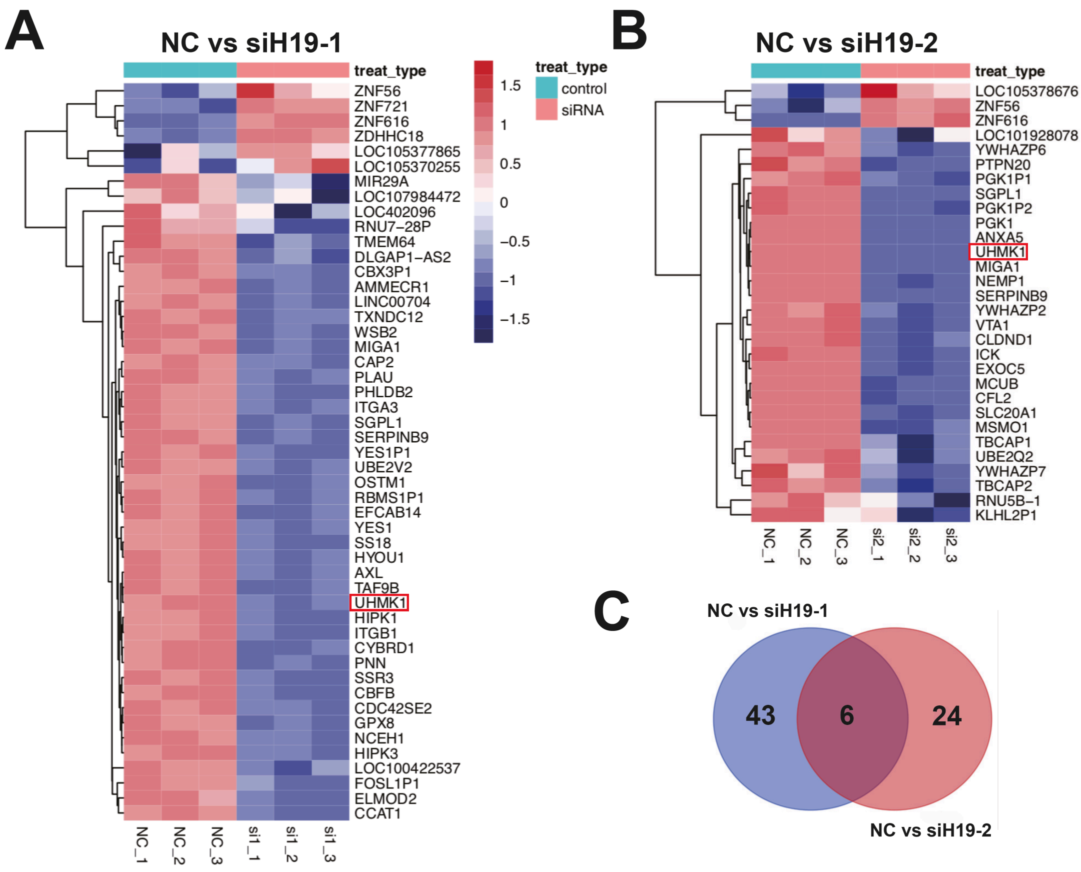

Figure S1 Selection of six lncRNA H19-induced genes. AllStar Negative Control siRHA (NC) or the siH19-1 and siH19-2 Flexible siRNA constructs were transfected into MIA-PaCa2 cells. Total RNA was harvested after 24 h followed by hybridization with a Clariom™ D Array, which was performed in triplicate. The threshold was set as fold change > 1 and the P value <0.05. (A) The heatmap represents significantly differentially expressed genes between the NC and siH19-1 groups. Red represents high expression, and blue represents low expression within a scale from 1.5 to -1.5 as indicated. (B) A heatmap representing significantly differentially expressed genes between the NC and siH19-2 groups in MIA-PaCa2 cells is shown. (C) Venn analysis was performed to compare and cross-validate the two microarrays, resulting in the selection of the six candidate genes.

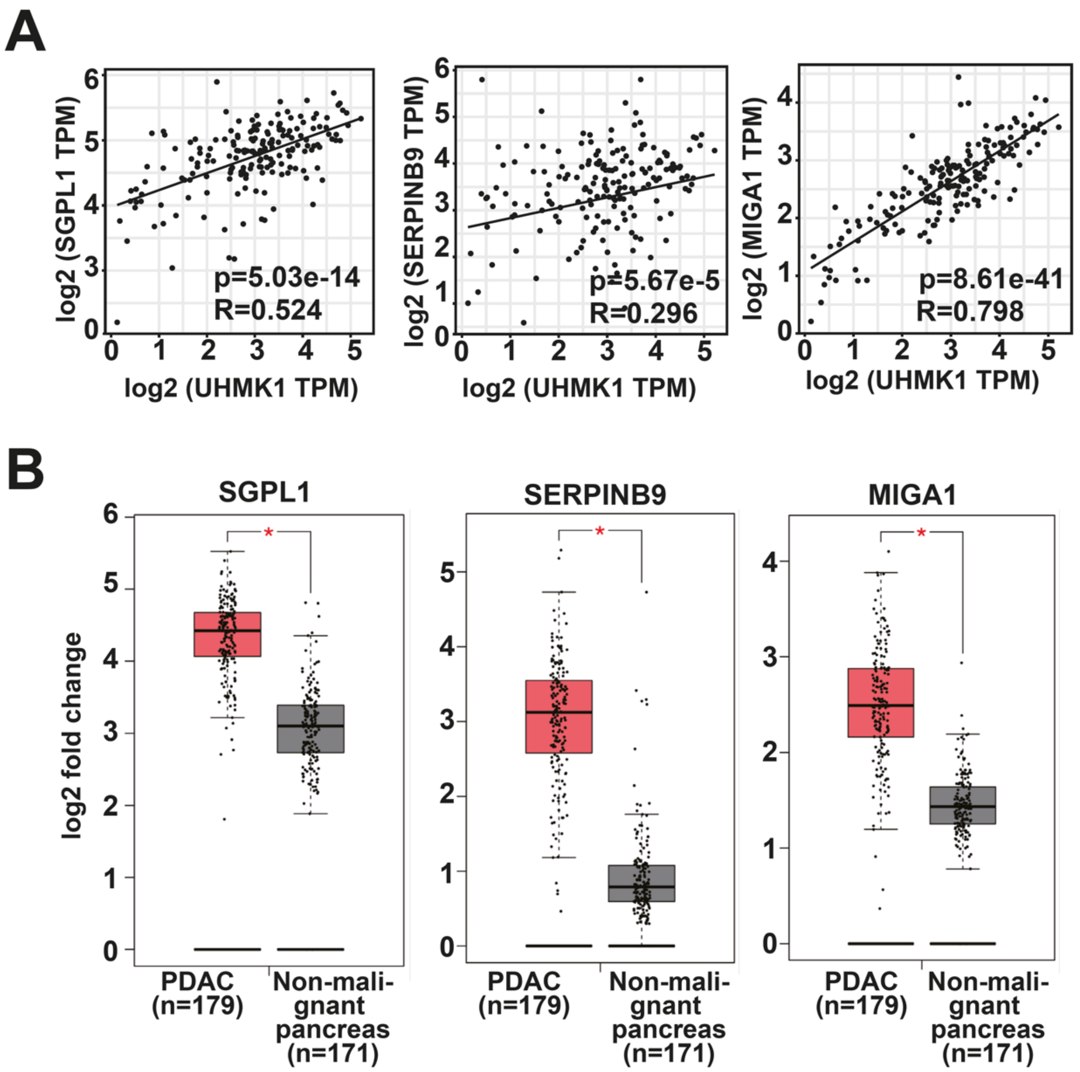

Suppl. Fig. S2. SGPL1, SERPINB9, and MIGA1 expression correlates with UHMK1 expression in PDAC tissue. (**A**) The TIMER 2.0 online tool was used to provide pairwise gene expression correlation analysis. The Pearson correlation coefficient (R) was detected between UHMK1 expression and SGPL1 expression (R=0.524), SERPINB9 expression (R=0.296), and MIGA1 expression (R=0.798). Transcripts per million (TPM). (**B**) The GEPIA online database was used to identify available expression data of the SGPL1, SERPIMB9, and MIGA1 genes in human PDAC and normal pancreatic tissues. The expression levels with the means ± SD are shown in the diagram. **P*<0.05. The cutoff of log_2-fold change was 1, and the cutoff for the P-value was 0.01. Log_2 (TPM+1) was used for log scale. Red columns represent PDAC tissue, and gray columns represent tissue from nonmalignant pancreas.

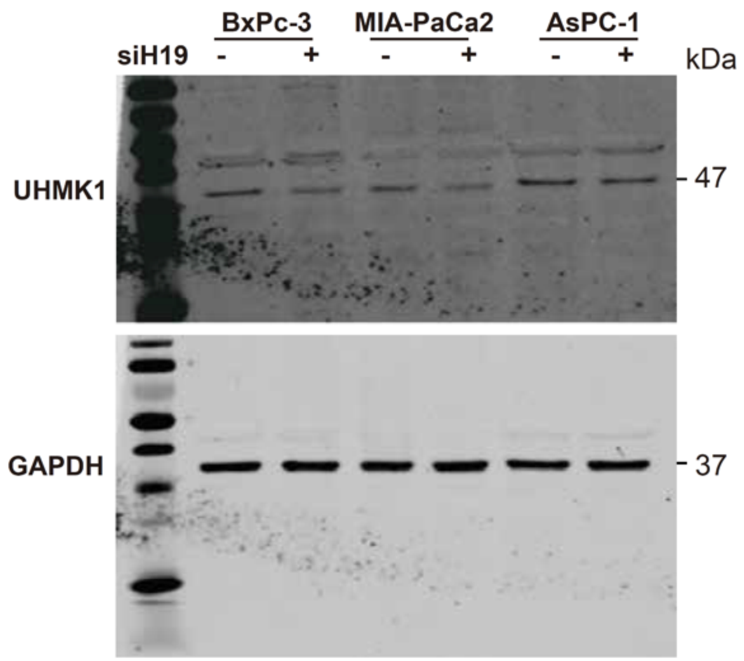

Figure S3. Original crude Western blot images. These images belong to Fig.1D and molecular weight markers in kilodalton (kDA) are shown. UHMK1 (47 kDa) was detected and GAPDH (37 kDa) was used as loading control.

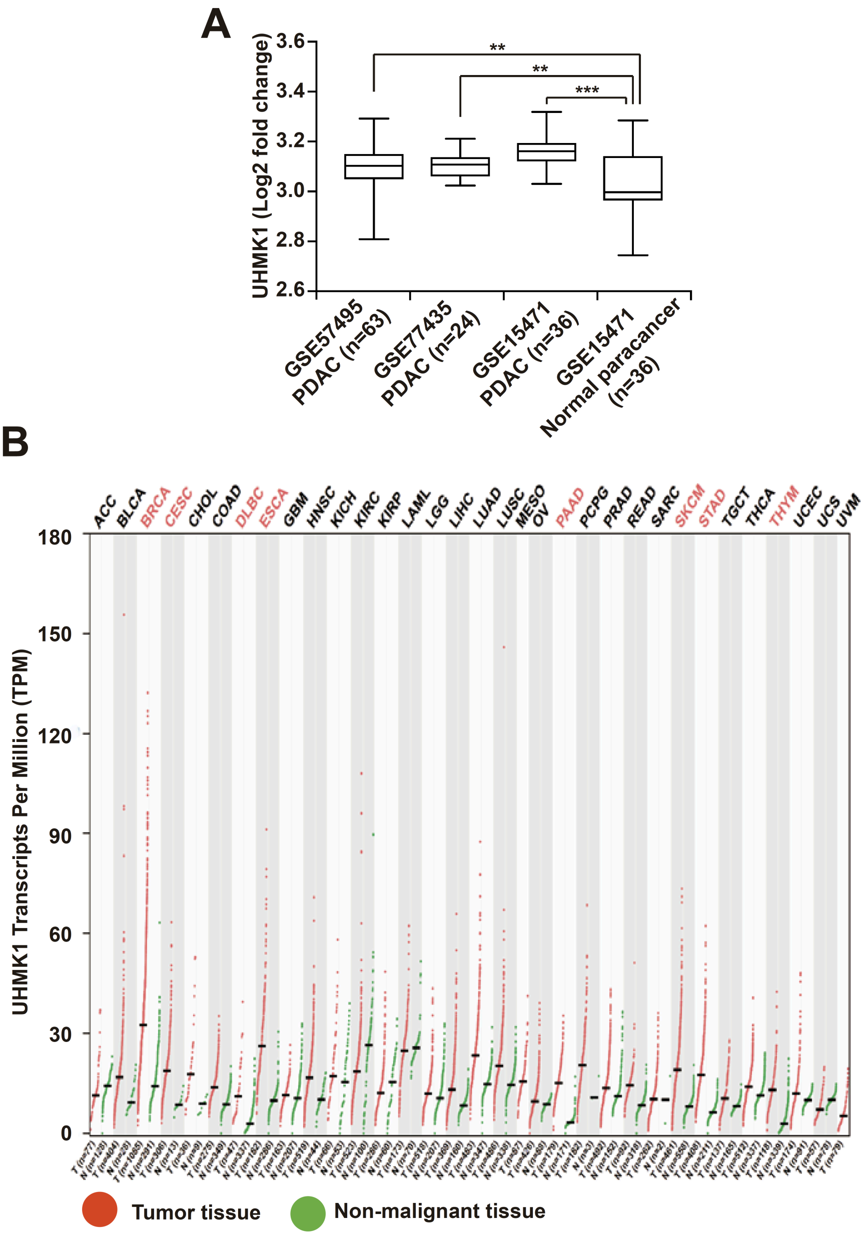

Figure S4 UHMK1 expression is upregulated in PDAC and other cancer types. (A) Three online datasets, GSE57492, GSE77435 and GSE15471, were extracted from the GEO database. UHMK1 expression was detected. (B) The expression of UHMK1 in different types of cancer was analyzed using the GEPIA database. Red dots indicate tumor tissue, and green dots indicate nonmalignant tissue. Red-marked cancer names on the top indicate statistically significant breast invasive carcinoma (BRCA), cervical squamous cell carcinoma and endocervical adenocarcinoma (CESC), lymphoid neoplasm diffuse large B-cell lymphoma (DLBC), esophageal carcinoma (ESCA), skin cutaneous melanoma (SKCM), stomach adenocarcinoma, and thymoma (THYM). **P*<0.05, ***P*<0.01, and ****P*<0.001.

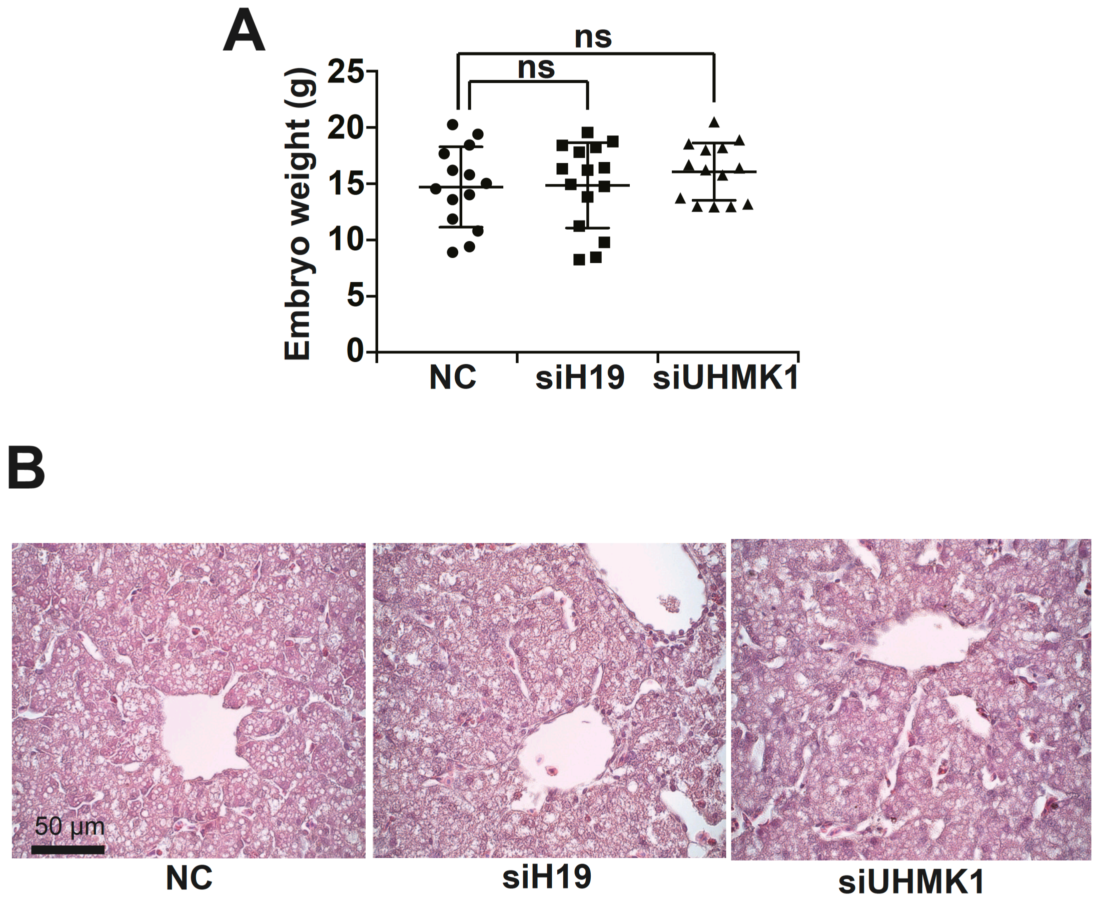

Figure S5 siH19 or siUHMK1 downregulation is not associated with side effects *in vivo*. (A) After resection of xenografts, the weight of each embryo was examined and is presented as a black dot in the diagram. The mean weights of each group are shown. (B) Representative H&E staining of embryonic frozen liver sections from each group is shown. ns: P >0.05.
